# Supplementary material for: PV-1: a novel molecular prognostic marker of distant metastases in various solid tumors
Source: EMBO Mol Med. 2025 Jul 21;17(9):2215–32. doi: 10.1038/s44321-025-00277-5 (PMC12423316; doi:10.1038/s44321-025-00277-5)
Supplement: Supplementary file 1 — Appendix [file 44321_2025_277_MOESM1_ESM.pdf]

Appendix

Table of contents

- Appendix Table S1
- Appendix Table S2
- Appendix Table S3
- Appendix Figure S1
- Appendix Figure S2

Appendix Table S1. Demographics and clinical characteristics of the STS patients (n=52).

		**Overall**	**Non-metastatic**	**Metastatic**	***P*-Value**	**Test**
n		52	24	28		
Female sex, n (%)		28 (53.8)	16 (66.7)	12 (42.9)	0.150	Chi-squared
Age, mean (SD)		54.9 (15.0)	53.2 (13.3)	56.3 (16.4)	0.465	t-test
Median percentage of PV-1+ cells, median [Q1,Q3]		20.0 [0.0,70.0]	10.0 [0.0,82.5]	25.0 [7.5,62.5]	0.765	Mann-Whitney
Follow-up time or metastasis onset (months), median [Q1,Q3]		74.0 [14.6,120.0]	120.0 [120.0,120.0]	17.1 [9.5,36.0]	**<0.001**	Mann-Whitney
Tumor subtype, n (%)	DDLPS	6 (11.5)	3 (12.5)	3 (10.7)	0.520	Chi-squared
	GIST	10 (19.2)	5 (20.8)	5 (17.9)		
	LMS	6 (11.5)	1 (4.2)	5 (17.9)		
	MPNST	6 (11.5)	3 (12.5)	3 (10.7)		
	MLPS	9 (17.3)	6 (25.0)	3 (10.7)		
	SFT	10 (19.2)	5 (20.8)	5 (17.9)		
	UPS	5 (9.6)	1 (4.2)	4 (14.3)		
Tumor grade, n (%)	low	32 (61.5)	16 (66.7)	16 (57.1)	0.676	Chi-squared
	high	20 (38.5)	8 (33.3)	12 (42.9)		
Tumor size (mm), mean (SD)		118.8 (71.5)	120.2 (84.2)	117.4 (57.7)	0.891	t-test

*Follow-up time refers to non-metastatic patients, while metastasis onset refers to metastatic patients. There were 4 missing data for the tumor size, which were imputed with the mean value of the other patients.

Appendix Table S2. Univariable and multivariable Cox PH regression analyses for STS patients (n=52; 28 metastatic; 24 non metastatic). Patients were stratified into high and low PV-1 groups (PV-1^{high} group n=27; PV-1^{low} group n=25).

	Univariable Cox PH model			Multivariable Cox PH model		
Variable	HR	(95% CI)	*p-value*	HR	(95% CI)	*p-value*
% PV-1+ cells[a]	1.00	(0.92–1.10)	0.96	—	—	—
PV-1 group[b]	1.34	(0.67–2.71)	0.41	—	—	—
Tumor size[c]	1.00	(0.96–1.04)	0.92	—	—	—
Tumor grade[d]	1.44	(0.71–2.93)	0.31	—	—	—
Female sex	0.48	(0.24-0.98)	**0.045**	0.48	(0.24-0.98)	**0.045**
Age[e]	1.11	(0.83–1.49)	0.47	—	—	—

PH, proportional hazards; HR, hazard ratio; CI, confidence interval.
[a] per 10-units increase
[b] high vs. low (reference)
[c] per 1-cm increase
[d] high vs. low (reference)
[e] per 10-years increase

Appendix Table S3. Univariable and multivariable Cox PH regression analyses for STS patients belonging to LMS, MPNST, SFT, and UPS histotypes (n=27; 17 metastatic, 10 non metastatic). Patients were stratified into high and low PV-1 groups (PV-1^{high} group n=18; PV-1^{low} group n=9).

	Univariable Cox PH model			Multivariable Cox PH model		
Variable	HR	(95% CI)	*p-value*	HR	(95% CI)	*p-value*
% PV-1+ cells[a]	1.04	(0.92–1.18)	0.49	—	—	—
PV-1 group[b]	3.59	(1.09–11.78)	**0.035**	3.86	(1.35-11.00)	**0.012**
Tumor size[c]	1.12	(1.05–1.19)	**<0.001**	1.13	(1.03–1.24)	**0.008**
Tumor grade[d]	1.40	(0.54–3.60)	0.48	—	—	—
Female sex	0.68	(0.26–1.74)	0.42	—	—	—
Age[e]	1.13	(0.80–1.60)	0.48	—	—	—

PH, proportional hazards; HR, hazard ratio; CI, confidence interval.
[a] per 10-units increase
[b] high vs. low (reference)
[c] per 1-cm increase
[d] high vs. low (reference)
[e] per 10-years increase

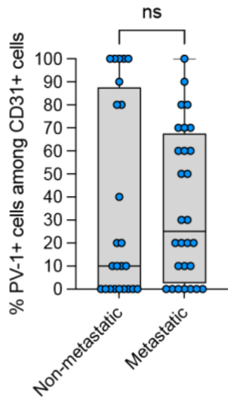

Appendix Figure S1. PV-1+ cell frequency in the primary tumor of 52 sarcoma patients from DDLPS, GIST, LMS, MLPS, MPNST, SFT, and UPS histotypes. Percentage of PV-1+ cells among CD31+ cells in the primary tumor of metastatic (n=28) and non-metastatic (n=24) sarcoma patients. Data are represented using box and whisker plots. Boxplots display values of minimum, first quartile, median, third quartile, and maximum. Each data point represents one sample. Statistical significance was evaluated using the two-sided Mann-Whitney unpaired test.

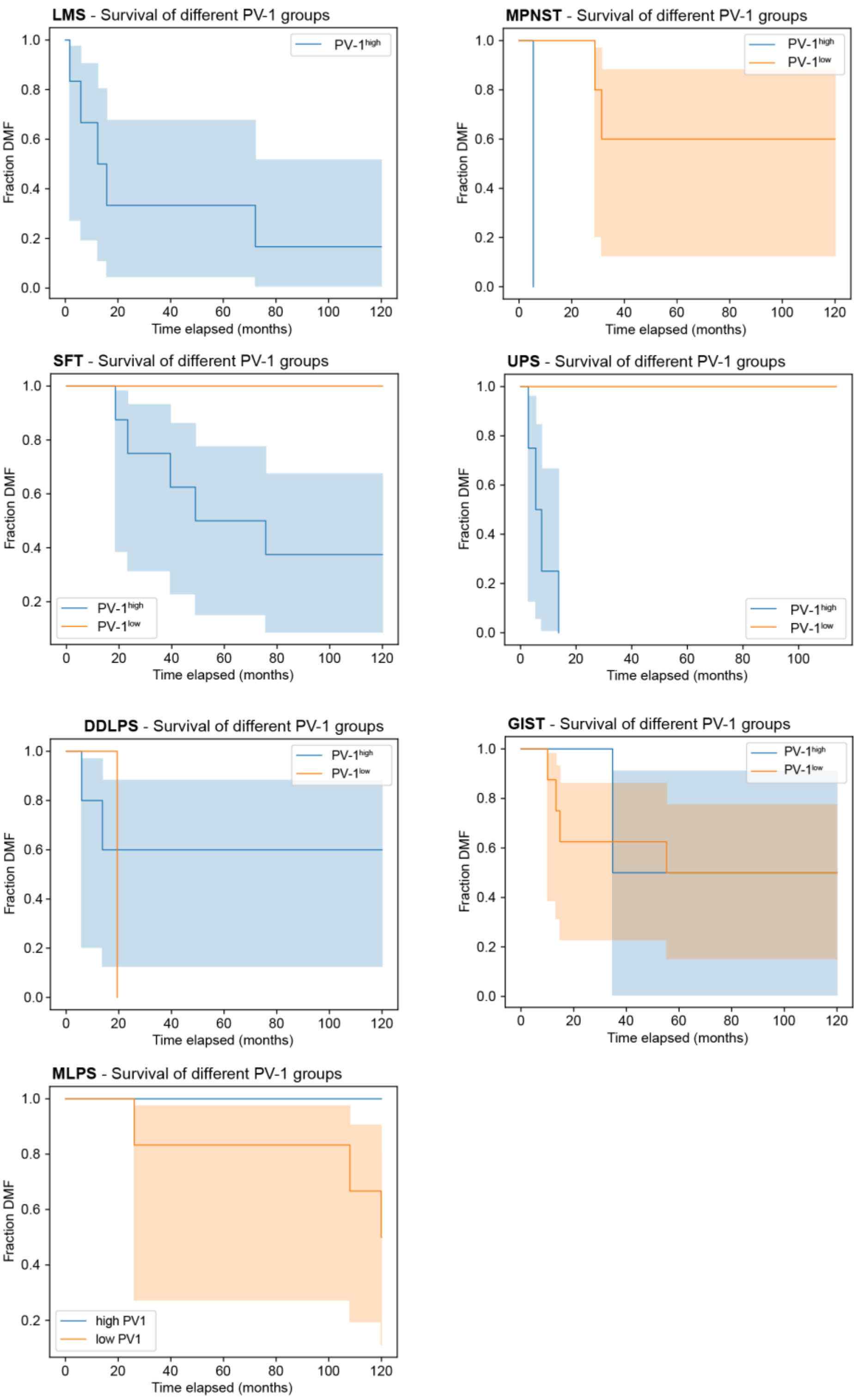

Appendix Figure S2. Distant-metastasis-free survival of sarcoma patients according to histotype and depending on the PV-1 group (high vs. low). PV-1^{high} group: % PV-1+/CD31+ cells ≥20; PV-1^{low}: % PV-1+/CD31+ cells <20. Leiomyosarcoma (LMS), n=6 (PV-1^{high}, n=6); Malignant peripheral nerve sheath tumor (MPNST), n=6 (PV-1^{high}, n=1); Solitary fibrous tumor (SFT), n=10 (PV-1^{high}, n=8); Undifferentiated pleomorphic sarcoma (UPS), n=5 (PV-1^{high}, n=4). Dedifferentiated liposarcoma (DDLPS), n=6 (PV-1^{high}, n=5); GIST, n=10 (PV-1^{high}, n=2); Myxoid liposarcoma (MLPS), n=9 (PV-1^{high}, n=3).
