# Supplementary material for: PV-1: a novel molecular prognostic marker of distant metastases in various solid tumors
Source: EMBO Mol Med. 2025 Jul 21;17(9):2215–32. doi: 10.1038/s44321-025-00277-5 (PMC12423316; doi:10.1038/s44321-025-00277-5)
Supplement: Supplementary file 7 — Expanded View Figures [file 44321_2025_277_MOESM7_ESM.pdf]

Expanded View Figures

Figure EV1. Time dependent ROC curves for luminal breast cancer at 5 years.

Plots of the time-dependent ROC analysis at 5 years performed for each of the clinical variables analyzed: tumor size, percentage of PV-1+ cells among CD31+ cells, Ki-67, age, number of positive lymph nodes, PgR, number of comorbidities, ER and tumor stage. $n = 30$ (metastatic, $n = 18$; non-metastatic, $n = 12$).

© The Author(s)

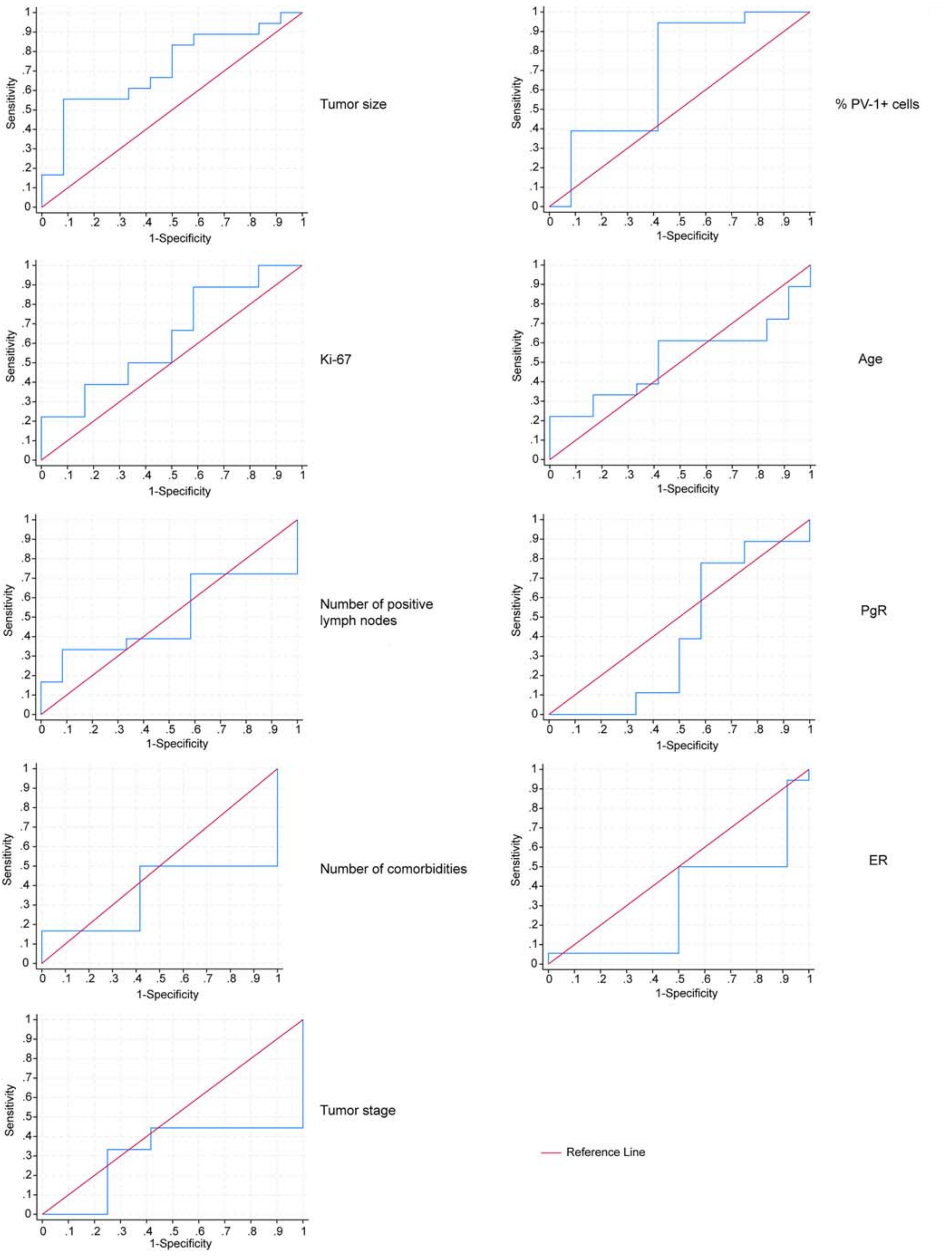
Tumor size
% PV-1+ cells
Ki-67
Age
Number of positive lymph nodes
PgR
Number of comorbidities
ER
Tumor stage
Reference Line
Sensitivity
1-Specificity

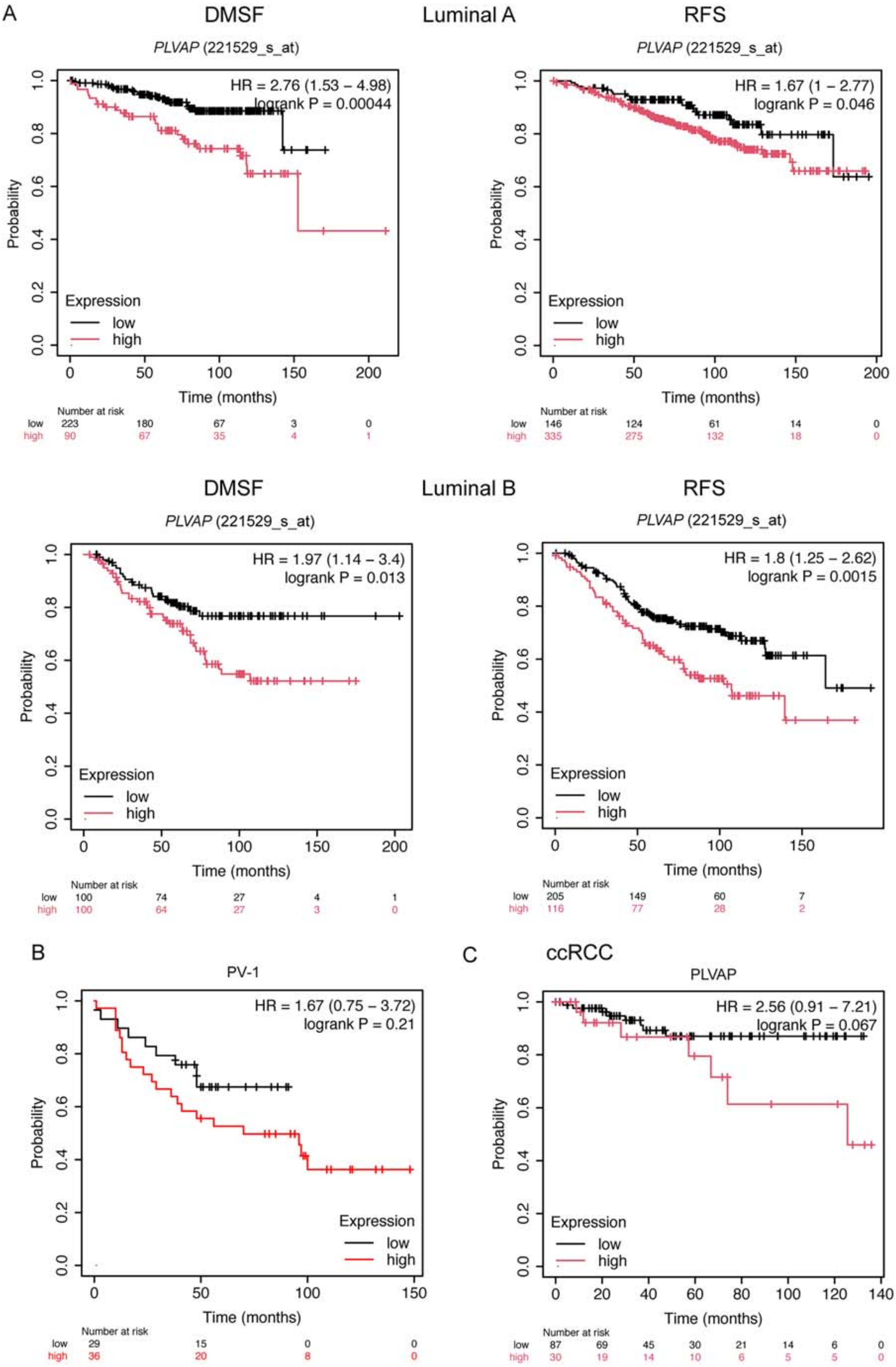
A
DMSF
Luminal A
RFS
PLVAP (221529_s_at)
HR = 2.76 (1.53 – 4.98)
logrank P = 0.00044
Probability
Expression
low
high
Time (months)
Number at risk
low 223 180 67 3 0
high 90 67 35 4 1
PLVAP (221529_s_at)
HR = 1.67 (1 – 2.77)
logrank P = 0.046
Number at risk
low 146 124 61 14 0
high 335 275 132 18 0
DMSF
Luminal B
RFS
PLVAP (221529_s_at)
HR = 1.97 (1.14 – 3.4)
logrank P = 0.013
Number at risk
low 100 74 27 4 1
high 100 64 27 3 0
PLVAP (221529_s_at)
HR = 1.8 (1.25 – 2.62)
logrank P = 0.0015
Number at risk
low 205 149 60 7
high 116 77 28 2
B
PV-1
HR = 1.67 (0.75 – 3.72)
logrank P = 0.21
Number at risk
low 29 15 0 0
high 36 20 8 0
C
ccRCC
PLVAP
HR = 2.56 (0.91 – 7.21)
logrank P = 0.067
Number at risk
low 87 69 45 30 21 14 6 0
high 30 19 14 10 6 5 5 0

Figure EV2. Kaplan–Meier survival analysis of PV-1 expression levels using the KM plotter tool.

(**A**) Distant-metastasis-free survival (DMSF) and relapse-free survival (RFS) were analyzed using mRNA gene chip data from Luminal A (DMSF, $n = 313$; RFS, $n = 481$) and Luminal B (DMSF, $n = 200$; RFS, $n = 321$) breast cancer patients treated with endocrine therapy. (**B**) Overall survival (OS) analysis based on PV-1 protein expression in breast cancer tissue ($n = 65$), including Luminal A, Luminal B, HER2-positive, and triple-negative breast cancer (TNBC) subtypes. (**C**) Relapse-free survival (RFS) was analyzed using mRNA-seq data from ccRCC patients ($n = 117$).

 © The Author(s)

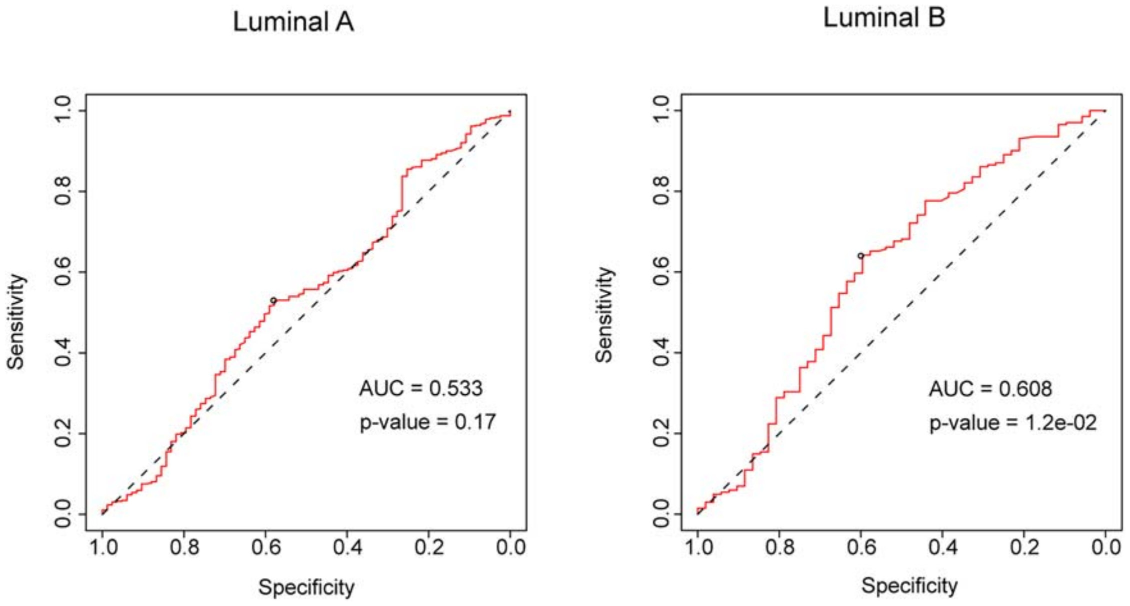

Figure EV3. ROC curve analysis of PV-1 expression in luminal breast cancer subtypes using ROC Plotter.

ROC Plotter constructs the ROC curve using 5-year relapse-free survival (RFS) as the clinical endpoint. Patients are classified based on clinical outcome: those without relapse within 5 years, and those with relapse within 5 years. PV-1 gene expression is evaluated as a continuous predictor of outcome. The analysis was performed separately for luminal A ($n = 637$; AUC = 0.533) and luminal B ($n = 253$; AUC = 0.608) breast cancer patients treated with endocrine therapy.

© The Author(s)

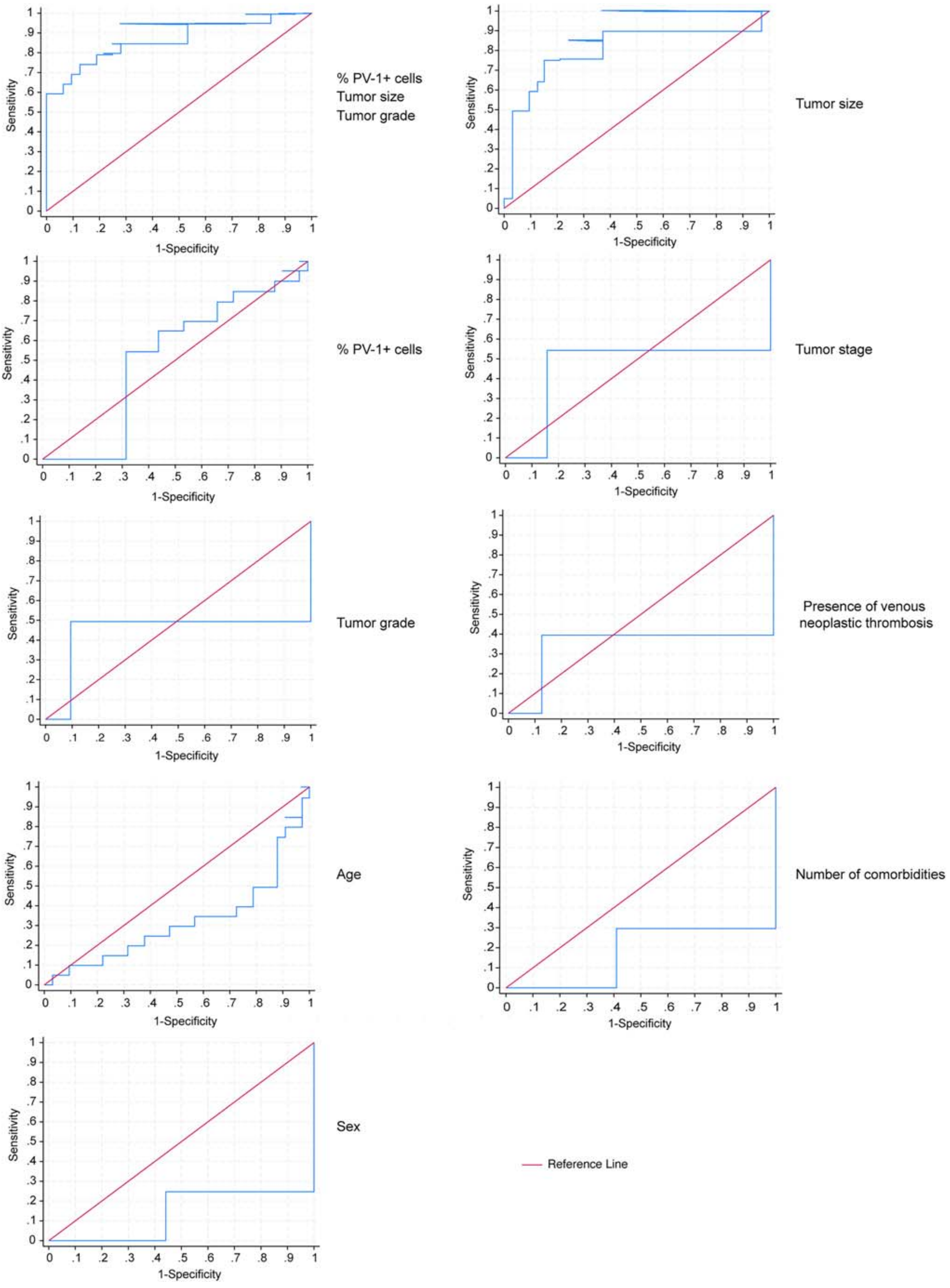
% PV-1+ cells
Tumor size
Tumor grade
Tumor size
% PV-1+ cells
Tumor stage
Tumor grade
Presence of venous neoplastic thrombosis
Age
Number of comorbidities
Sex
Reference Line
Sensitivity
1-Specificity

Figure EV4. Time-dependent ROC curves for ccRCC at 5 years.

Plots of the time-dependent ROC analysis at 5 years performed for each of the clinical variables analyzed (tumor size, percentage of PV-1+ cells among CD31+ cells, tumor stage, tumor grade, presence of venous neoplastic thrombosis, age, number of comorbidities and sex) and for the combination of tumor size, tumor grade, and percentage of PV-1+ cells. $n = 52$ (metastatic, $n = 22$; non-metastatic, $n = 30$).

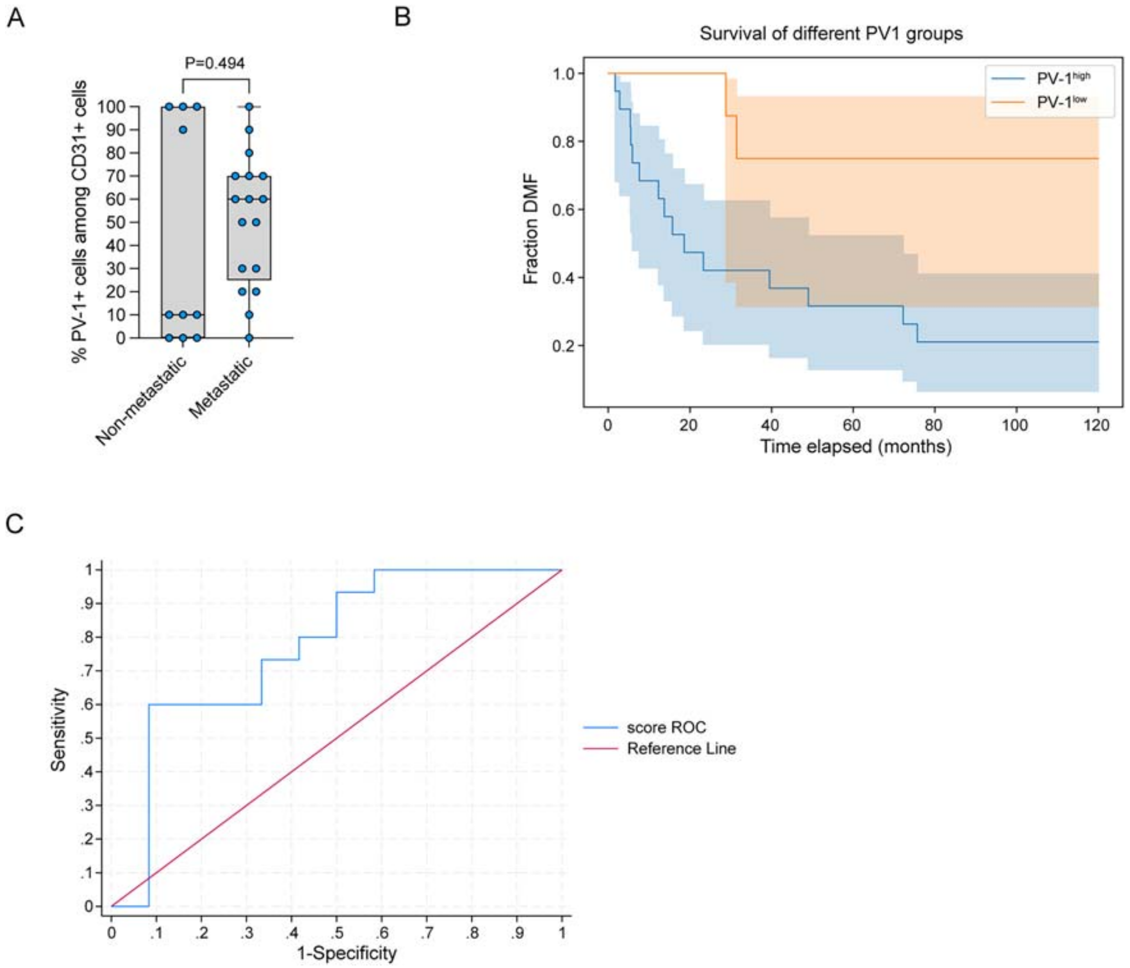

Figure EV5. PV-1+ cell frequency in the primary tumor of metastatic and non-metastatic sarcoma patients from the LMS, MPNST, SFT, and UPS histotypes.

(**A**) Percentage of PV-1+ cells among CD31+ cells in the primary tumor of metastatic ($n = 17$) and non-metastatic ($n = 10$) sarcoma patients. Data are represented using box and whisker plots. Boxplots display values of minimum, first quartile, median, third quartile, and maximum. Each data point represents one sample. Statistical significance was evaluated using the two-sided Mann–Whitney unpaired test. (**B**) Metastasis-free survival of sarcoma patients depending on the PV-1 group (high vs. low). PV-1^{high} group: % PV-1+/CD31+ cells ≥20; PV-1^{low}: % PV-1+/CD31+ cells <20. $n = 27$ (metastatic, $n = 17$; non-metastatic, $n = 10$). (**C**) Plot of the time-dependent ROC analysis at 5 years performed for the combination of PV-1 group and tumor size (score). $n = 27$ (metastatic, $n = 17$; non-metastatic, $n = 10$).

 © The Author(s)
